# Leukocytes from Patients with Drug-Sensitive and Multidrug-Resistant Tuberculosis Exhibit Distinctive Profiles of Chemokine Receptor Expression and Migration Capacity

**DOI:** 10.1155/2021/6654220

**Published:** 2021-04-21

**Authors:** Ranferi Ocaña-Guzmán, Norma A. Téllez-Navarrete, Lucero A. Ramón-Luing, Iliana Herrera, Marlon De Ita, José-Luis Carrillo-Alduenda, José Alberto Choreño-Parra, Karen Medina-Quero, Joaquín Zúñiga, Leslie Chávez-Galán

**Affiliations:** ^1^Laboratory of Integrative Immunology, Instituto Nacional de Enfermedades Respiratorias “Ismael Cosío Villegas”, Mexico City, Mexico; ^2^Laboratory of Cellular Biology, Instituto Nacional de Enfermedades Respiratorias “Ismael Cosío Villegas”, Mexico City, Mexico; ^3^Unit of Medical Research in Human Genetics, Hospital de Pediatría, Centro Médico Nacional Siglo XXI, Mexico City, Mexico; ^4^Sleep Medicine Unit, Instituto Nacional de Enfermedades Respiratorias “Ismael Cosío Villegas”, Mexico City, Mexico; ^5^Laboratory of Immunobiology and Genetics, Instituto Nacional de Enfermedades Respiratorias “Ismael Cosío Villegas”, Mexico City, Mexico; ^6^Laboratory of Immunology, Escuela Militar de Graduados de Sanidad, Mexico City, Mexico; ^7^Escuela de Medicina y Ciencias Médicas, Tecnológico de Monterrey, Mexico City, Mexico

## Abstract

Tuberculosis (TB), caused by *Mycobacterium tuberculosis* (Mtb), remains as a leading infectious cause of death worldwide. The increasing number of multidrug-resistant TB (MDR-TB) cases contributes to the poor control of the TB epidemic. Currently, little is known about the immunological requirements of protective responses against MDR-TB. This is of major relevance to identify immune markers for treatment monitoring and targets for adjuvant immunotherapies. Here, we hypothesized that MDR-TB patients display unique immunophenotypical features and immune cell migration dynamics compared to drug-sensitive TB (DS-TB). Hence, we prospectively conducted an extensive characterization of the immune profile of MDR-TB patients at different time points before and after pharmacological therapy. For this purpose, we focused on the leukocyte expression of chemokine receptors, distribution of different monocyte and lymphocyte subsets, plasma levels of chemotactic factors, and *in vitro* migration capacity of immune cells. Our comparative cohort consisted of DS-TB patients and healthy volunteer donors (HD). Our results demonstrate some unique features of leukocyte migration dynamics during MDR-TB. These include increased and prolonged circulation of CD3+ monocytes, CCR4+ monocytes, EM CD4+ T cells, EM/CM CD8+ T cells, and CXCR1+CXCR3+ T cells that is sustained even after the administration of anti-TB drugs. We also observed shared characteristics of both MDR-TB and DS-TB that include CCR2+ monocyte depletion in the blood; high plasma levels of MPC-1, CCL-7, and IP-10; and increased responsiveness of leukocytes to chemotactic signals *in vitro*. Our study contributes to a better understanding of the MDR-TB pathobiology and uncovers immunological readouts of treatment efficacy.

## 1. Introduction

*Mycobacterium tuberculosis* (Mtb), the causative agent of tuberculosis (TB), is a leading infectious cause of mortality worldwide. In 2018, the World Health Organization (WHO) estimated that 10 million new TB cases and 1.2 million deaths occurred globally [1]. Most infected individuals carried Mtb strains sensitive to first-line drugs (DS-TB). However, during the same year, the WHO reported half a million new multidrug-resistant TB (MDR-TB) cases, from which only 186,772 were officially notified and treated [1]. Besides novel pharmacological regimens, a better understanding of the immunopathogenesis of MDR-TB is urgently required. As such, the exploration of immunological differences between MDR-TB and DS-TB may reveal unique characteristics that could serve as indicators of treatment efficacy or targets for new immunotherapies against MDR-TB.

After reaching the lower respiratory tract, Mtb gets into contact with phagocytes, which respond by producing cytokines and chemokines. These molecules mediate the recruitment of other leukocyte subsets, thus promoting the formation of multicellular immune structures termed granulomas [2]. T cells arrive at forming granulomas and are necessary to maintain both their structure and protective functions. Indeed, T cells sustain the bactericidal activity of macrophages located within granulomas via the production of interferon-gamma (IFN-*γ*) and tumor necrosis factor-alpha (TNF-*α*) [3, 4].

Immune cell traffic is regulated by different chemokine/chemokine receptor axes. For instance, the monocyte chemoattractant protein 1 (MCP-1) and the C-C motif chemokine ligand 17 (CCL-17) exert monocyte recruitment through their interaction with the C-C-motif chemokine receptor 2 (CCR2) and 4 (CCR4) [5]. A CCR4 deficiency or increased frequency of CCR2+ cells are predictors of active TB and correlate with high Mtb burden, whereas MCP-1 levels serve as a biomarker to differentiate active from latent TB infection [6–10]. Moreover, CCR2 has shown a crucial role in mediating alveolar macrophage migration during granuloma formation [11]. Recently, a previously unrecognized myeloid cell subpopulation expressing the molecule CD3 has been described. These myeloid CD3+ cells produce proinflammatory cytokines and accumulate within Mtb infection sites. However, the chemotactic signals promoting their recruitment, including the classic CCR2 pathway, are unknown [12, 13].

CXCR3-, CCR5-, and CCR6-expressing T cells migrate into the Mtb-infected lung in response to interleukin 23 (IL-23) and interleukin 17 (IL-17), where they induce the activation of macrophages [14–19]. Interferon *γ*-induced protein 10 (IP-10) and interleukin 8 (IL-8), ligands of CXCR3 and CXCR1, respectively, have also been implicated in the immune system reactions against Mtb. Indeed, total blood cells from active TB patients show a higher CXCR1 expression than healthy individuals and latent TB-infected subjects [20–22].

MDR-TB strains are thought to differentially regulate immunity compared with DS-TB. Indeed, *in vitro* studies have found that MDR-TB strains damper the cytotoxic activity and chemokine production capacity of CD8+ T cells, as well as IL-8 and TNF-*α* release by lung epithelial cells [23, 24]. Furthermore, MDR-TB strains exhibit changes in their cell walls' lipid composition, which induce a metabolic shift in phagocytes and impair leukocytes' recruitment to the infected lungs in mice [25]. Hence, MDR-TB strains may also induce different dynamic profiles of leukocyte migration leading to a delayed control of the infection in humans.

In this work, we hypothesized that MDR-TB patients display phenotypical changes in chemokine receptors' leukocyte expression compared to DS-TB individuals. Consequently, their immune cell migration dynamics must be different and normalized after anti-MDR-TB therapy. Therefore, we prospectively evaluated the frequency and distribution of several immune cell subpopulations expressing different chemokine receptors in the peripheral blood of DS-TB and MDR-TB patients. Also, we analyze their migration capacity before and after anti-TB treatment.

Our results demonstrate some unique features of leukocyte migration dynamics during MDR-TB. These include increased and prolonged circulation of CD3+ monocytes, CCR4+ monocytes, EM CD4+ T cells, EM/CM CD8+ T cells, and CXCR1+CXCR3+ T cells that is sustained even after the administration of anti-TB drugs. We also observed shared characteristics of both MDR-TB and DS-TB that include depletion of CCR2+ monocytes from the blood; high plasma levels of MPC-1, CCL-7, and IP-10; and increased responsiveness of leukocytes to chemotactic signals *in vitro*. Our study contributes to a better understanding of the pathobiology of MDR-TB and uncovers immunological readouts that could serve as biomarkers for treatment monitoring.

## 2. Materials and Methods

### 2.1. Study Population

We conducted a prospective study in patients admitted to the TB Clinic of the Instituto Nacional de Enfermedades Respiratorias Ismael Cosío Villegas (INER) in Mexico City between 2011 and 2014. Pulmonary TB diagnosis relied on combined clinical criteria, chest X-ray images, acid-fast bacilli smear-positive staining, and Mtb-positive cultures in sputum samples [26]. Eligible patients should not have received anti-TB drugs at enrollment.

Participants were divided into two groups according to their drug-sensitivity status. The first group was composed of DS-TB patients with no previous TB history who provided peripheral blood samples at baseline and at 1 and 6 months after anti-TB treatment (moTBt). These time points correspond to the initiation of the intensive and the end of the maintenance phases of treatment, respectively. The second group included MDR-TB patients with a previous diagnosis of TB resistant to both rifampicin and isoniazid. These individuals provided blood samples at baseline and at 1, 3, 8, and 16 moTBt, which correspond to the initial, intensive, and end of the maintenance treatment phases.

In most cases, the follow-up was carried out by the INER clinician staff, who verified treatment efficacy through the negative conversion of sputum cultures. Clinical monitoring of MDR-TB patients included consecutive monthly sputum cultures. Notably, as our institution is a national reference center, patients from places distant to Mexico City were referred to their local health care centers (LHCC) after obtaining negative culture results. Thus, some participants completed their treatment at LHCC and were lost from the initial cohorts.

A third group of asymptomatic healthy volunteer donors (HD) without a history of contact with TB patients were enrolled and served as controls. These individuals received the *Mycobacterium bovis* Bacillus Calmette-Guérin (BCG) vaccine at birth and had a negative tuberculin skin test. Subjects with comorbidities, pulmonary cancer, autoimmune conditions, and those with coinfections, including the human immunodeficiency virus (HIV), were ineligible.

### 2.2. Demographic and Clinical Characteristics

Clinicians retrieved demographic data from DS-TB and MDR-TB patients by direct interview and clinical evaluation. Laboratory parameters routinely quantified in each patient's blood as part of their integral clinical follow-up, including hemoglobin, glucose, and blood cell counts, were obtained from electronic medical records.

### 2.3. Flow Cytometry

Peripheral blood mononuclear cells (PBMCs) were obtained from total peripheral blood samples of recruited patients using a Ficoll density gradient (Lymphoprep™; Axis-Shield, Oslo, Norway). After collection, PBMCs were counted by Trypan blue exclusion to determine their viability. Plasma aliquots from each patient were stored at -80°C until used. For phenotypical analysis of PBMCs, the cells were incubated with fluorochrome-conjugated monoclonal antibodies (mAbs) against CD3, CD4, CD8, CD14, CD11b, CD16, CD45RA, CCR2, CCR4, CCR7, CXCR1, and CXCR3 (BioLegend, San Diego, CA, USA; Supplementary Table S1) for 20 minutes at 4°C. Then, cells were washed and resuspended in a staining buffer (BioLegend, San Jose, CA, USA) before FACS analysis.

Multiparametric flow cytometry was performed using a FACS Aria II flow cytometer (Becton Dickinson, San Jose, CA, USA). Immune cell subpopulations were gated based on their forward and side scatter characteristics and different markers' expression. The side/forward scatter gating strategy was used to exclude dead cells. Fluorescence minus one (FMO) controls were employed to set the gates for specific immune cell subpopulations. We acquired at least 100,000 events per sample. The cells utilized for FMO were stained and acquired in parallel. The compensation set-up and the calculation of the frequency of specific cell subsets were conducted using FlowJo software (FlowJo, LLC, Ashland, OR, USA).

### 2.4. Cytokine and Chemokine Quantification

Plasma levels of MCP-1, CCL-17, IP-10, and IL-8 were quantified by enzyme-linked immunosorbent assays (ELISA) following the manufacturer's protocols (BioLegend, San Jose, CA, USA).

### 2.5. Migration Assays

Migration assays were performed using different concentrations of MCP-1 (5, 10, 30, 50, and 100 ng/ml), CCL-17 (0.5, 1, 5, 10, and 20 ng/ml), IP-10 (5, 10, 20, 30, and 50 ng/ml), and IL-8 (10, 30, 50, 100, and 150 ng/ml) following the manufacturer's protocols (Supplementary Figure S1). Briefly, the chemoattractant factors (Peprotech) were diluted in 150 *μ*l of RPMI-1640 medium and added to the bottom well (feeder tray) of a 96-well chemotaxis plate (Cytoselect 96-well cell migration assay-fluorometric format, Cell Biolabs, Inc.). PBMCs were resuspended in RPMI-1640 serum-free medium at a concentration of 2.0 × 10^6^ cells/ml, and 100 *μ*l was added on the 5 *μ*m pore size chamber's membrane (migration plate) for 4 h at 37°C in 5% CO_2_. Then, cells that did not migrate were removed from the top chamber's membrane by inversion, and those attached to the membrane were recovered using 150 *μ*l of cell detachment solution. Afterward, 75 *μ*l of this elute (detached cells) was transferred onto a clean 96-well plate and combined with 75 *μ*l of the cells that had migrated to the chemoattractant solution (feeder tray). Cells were lysed, and the total number of transmigrated cells was quantified using CyQuant® GR Dye (Synergy™ HT; BioTek, Inc., USA). Feeder tray wells with RPMI-1640+BSA 1% were employed as controls for each chemokine. The remaining cells (150 *μ*l) were analyzed through flow cytometry. Migration experiments were performed by triplicate using PBMCs from three DS-TB and three MDR-TB patients obtained before and after treatment.

### 2.6. Ethical Approval

The Institutional Review Board of the INER in Mexico City approved the study (protocol number: C44-11). Written informed consent was obtained from all study participants or their legal guardians. All patients received anti-TB chemotherapy following the guidelines of the Mexican Ministry of Health. All laboratory procedures were performed in agreement with the 1964 Helsinki Declaration and the ethical standards of the Institutional Ethics Committee.

### 2.7. Statistical Analysis

Data analysis was performed using GraphPad Prism 8 (GraphPad Software, La Jolla, CA, USA) and Stata/SE 13.0 (StataCorp LLC, College Station, TX, USA). Data are presented as means with the standard error of the mean (SEM) or medians with interquartile ranges (IQR). Differences between the two groups were analyzed using the Mann–Whitney *U* test. For comparisons between more than two groups, we used multiple Student's *t*-tests, and the *p* values were corrected for multiple comparisons, utilizing the Sidak–Bonferroni's method.

## 3. Results

### 3.1. Participant Characteristics

**Figure 1:**
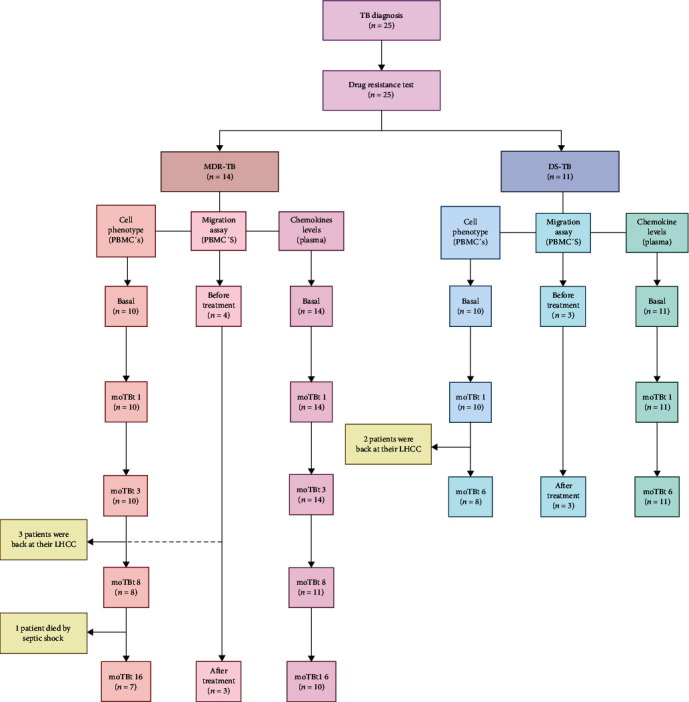
Workflow of DS-TB and MDR-TB patient recruitment and follow-up. A total of 25 TB patients were enrolled, 14 with multidrug-resistant (MDR-TB) and 11 with drug-sensitive (DS-TB). The first group was maintained at baseline, 1 month, and 3 months of anti-TB therapy (moTBt). Then, three patients were sent back to their local health care center (LHCC), and the number of patients decreased to 11 at 8 moTBt. Furthermore, one patient died of septic shock. Thus the MDR-TB cohort at 16 moTBt included 10 patients (left). The cohort of DS-TB patients was also maintained from baseline to 1 moTBt, but at 3 moTBt, two patients were sent back to their LHCC. Thus, the DS-TB cohort at 6 moTBt included 9 patients (right). Cells from two DS-TB patients were used to cell phenotype and migration assay because of the low number of cells yielded.

We enrolled 25 TB patients: 11 with DS-TB and 14 with MDR-TB. The DS-TB cohort was maintained over the first two time points of evaluation (baseline and 3 moTBt). Then, two patients were sent back to their LHCC, and the cohort was reduced to 11 DS-TB patients by the 6 moTBt (Figure 1). Cells from two DS-TB patients were included in the phenotype and migration assay analyses due to the low cell number yielded. On the other hand, by the 8 moTBt, three patients in the MDR-TB group were sent back to their LHCC (*n* = 11). Also, one MDR-TB patient died of septic shock, and the cohort decreased to 10 patients at 16 moTBt (Figure 1).

The clinical and demographic characteristics of study participants are summarized in Table 1. Twenty-one participants (84%) were male with a median age of 41 years (31-47 years, IQR). Also, 15 patients (60%) were diagnosed with type 2 diabetes mellitus (DM2). Both participant groups showed similar clinical and laboratory characteristics at baseline (Tables 1 and 2), except that all MDR-TB patients reported a history of previous TB treatment two to eight years ago. However, only one DS-TB patient referred having had TB previously (Table 1). Six HD were recruited and served as controls. Although the HD group was younger than TB patients, the difference was not statistically significant (Table 1).

### 3.2. CD3+ Monocytes Are Increased during DS-TB and MDR-TB

**Figure 2:**
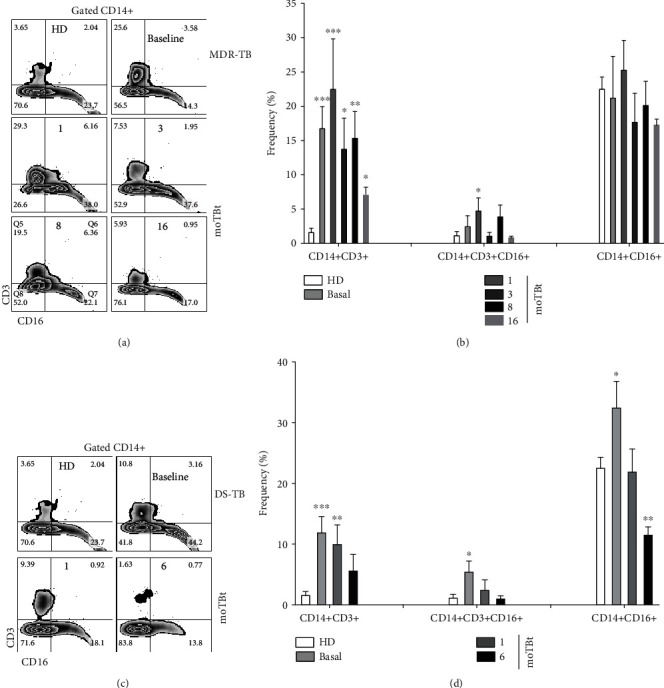
Increased frequency of CD14+CD3+ monocytes during DS-TB and MDR-TB. Representative zebra plots illustrating the gating strategy used to analyze CD3 and CD16 expressions in total CD14+ cells. Peripheral blood mononuclear cells (PBMCs) from multidrug-resistant tuberculosis (MDR-TB) and drug-sensitive tuberculosis (DS-TB) patients were obtained at different time points and employed for phenotypical characterization by flow cytometry (a and c, respectively). (b) Frequency of CD14+CD3+, CD14+CD3+CD16+, and CD14+CD16+ subpopulations in MDR-TB patients (HD: *n* = 6; MDR-TB: baseline and 1 and 3 moTBt *n* = 10, 8 moTBt *n* = 8, 16 moTBt *n* = 7) (d) Frequency of CD14+CD3+, CD14+CD3+CD16+, and CD14+CD16+ subpopulations in DS-TB patients (HD: *n* = 6; DS-TB: baseline and 1 moTBt *n* = 10, 6 moTBt *n* = 8). Bar graphs show means ± standard error of the mean (SEM). Differences between patient groups and HD at each time point were analyzed using multiple Student's *t*-test corrected for multiple comparisons using the Sidak–Bonferroni method. ^∗^*p* < 0.05, ^∗∗^*p* < 0.01, ^∗∗∗^*p* < 0.001, and ^∗∗∗∗^*p* < 0.0001. moTBt: months of antituberculosis treatment.

Our first approach was to analyze the inflammatory status of monocytes in DS-TB and MDR-TB patients. Circulating monocytes are the primary precursor of tissue-resident macrophages. Classical monocytes (CD14+CD16-) constitute the most abundant subtype in the human blood, but the CD14+CD16+ subset has the characteristic of secreting high amounts of proinflammatory cytokines such as TNF-*α* and interleukin 1 beta (IL-1*β*) [27, 28]. A novel macrophage subpopulation expressing CD3 also delivers proinflammatory cytokines at Mtb infection sites [12, 29]. Using flow cytometry, we analyzed total CD14+ cells to determine whether the proinflammatory CD3+ monocyte subset is the same as the CD16+ subpopulation (Figures 2(a) and 2(c)).

Interestingly, we observed that CD14+CD3+ and CD14+CD16+ monocytes are independent subsets. In fact, from the total monocyte population of MDR-TB patients, 25% were single CD3+, 25% were single CD16+, and only 5% coexpressed CD3 and CD16 (Figure 2(b)). Overall, CD14+CD3+ monocytes were found increased in both DS-TB and MDR-TB as compared to HD. However, MDR-TB patients showed a higher frequency of CD14+CD3+ monocytes at baseline than DS-TB (25% vs. 13%, respectively), although the differences were not significant. Furthermore, CD14+CD3+ monocytes remained high in MDR-TB, even at 16 moTBt, whereas they decreased in DS-TB at 6 moTBt (Figures 2(b) and 2(d)). These data demonstrate that CD3+ monocytes are an independent myeloid subpopulation increased during TB and maintained for extended periods among MDR-TB patients.

### 3.3. Expression of CCR2 and CCR4 in Monocytes from DS-TB and MDR-TB Patients

**Figure 3:**
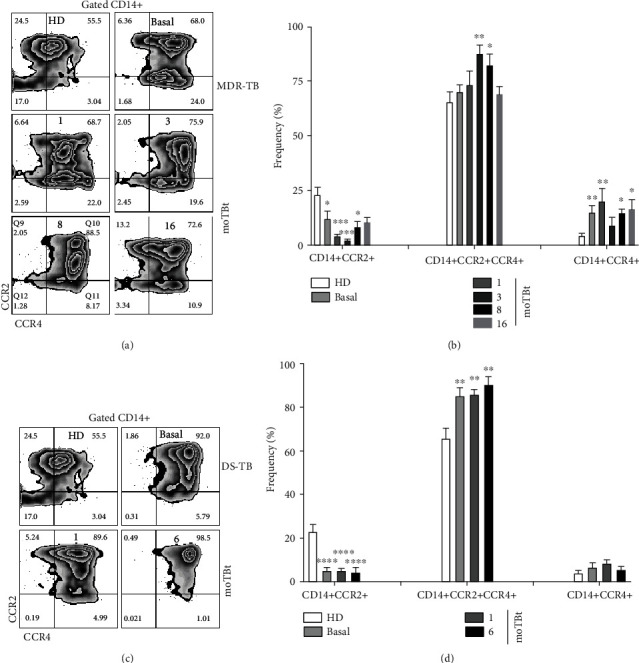
Differential expression of CCR2 and CCR4 in peripheral blood monocytes from MDR-TB and DS-TB patients. Representative zebra plots showing the strategy of analysis used to evaluate CCR2 and CCR4 expression in total CD14+ monocytes by flow cytometry. Peripheral blood mononuclear cells (PBMCs) from multidrug-resistant (MDR-TB) or drug-sensitive (DS-TB) patients were obtained at different time points before and after anti-TB treatment and employed for phenotypical characterization (a and c, respectively). (b) Frequency of CD14+CCR2+, CD14+CCR2+CCR4+, and CD14+CCR4+ subpopulations in MDR-TB patients (HD: *n* = 6, MDR-TB: basal, 1, and 3 moTBt *n* = 10, 8 moTBt *n* = 8, and 16 moTBt *n* = 7). (d) Frequency of CD14+CCR2+, CD14+CCR2+CCR4+, and CD14+CCR4+ subpopulations in DS-TB patients (HD *n* = 6; DS-TB: basal, and 1 moTBt *n* = 10, and 6 moTBt *n* = 8). Bar graphs display means ± standard error of the mean (SEM). Differences between patient groups and HD at each time point were analyzed using multiple Student's *t*-test corrected for multiple comparisons using the Sidak–Bonferroni method. ^∗^*p* < 0.05, ^∗∗^*p* < 0.01, ^∗∗∗^*p* < 0.001, and ^∗∗∗∗^*p* < 0.0001. moTBt: months of antituberculosis treatment.

Migration of immune cells to the sites of Mtb infection is crucial to mount protective immune responses. CCR2 is an essential chemokine receptor that favors monocyte chemotaxis [5]. We speculated that immune cell traffic kinetics are modified during MDR-TB, which could be partially related to different leukocyte chemokine receptor expression patterns. Using flow cytometry, we looked for phenotypical changes in the expression of CCR2 and CCR4 in peripheral blood CD14+ monocytes from DS-TB and MDR-TB patients (Figures 3(a) and 3(c)).

Strikingly, CD14+CCR2+ monocytes were depleted from the circulation of MDR-TB patients with respect to HD at baseline and further decreased until 8 moTBt. However, this population returned to baseline frequencies similar to HD at 16 moTBt (Figure 3(b)). In contrast, CD14+CCR4+ and CD14+CCR2+CCR4+ monocytes were found elevated during follow-up, but only CD14+CCR4+ monocytes continued increasing even at 16 moTBt in MDR-TB patients (Figure 3(b)). Regarding DS-TB patients, we found a similar depletion of CD14+CCR2+ monocytes that, contrary to MDR-TB patients, was not recovered at the end of therapy. Additionally, DS-TB patients showed enrichment of CD14+CCR2+CCR4+ in the blood, although single-positive CD14+CCR4+ monocytes did not increase as observed in individuals with MDR-TB (Figure 3(d)). We also evaluated which monocyte subpopulation was expressing more CCR2 (Supplementary Figure S2). Interestingly, classical monocytes were the predominant CCR2+ monocyte subpopulation in MDR-TB patients and normalized at 8 moTBt (Supplementary Figure S2). Meanwhile, classical and CD16+ monocytes subpopulations from DS-TB expressed high CCR2 levels even at 6 moTBt (Supplementary Figure 2). Of note, CD3+ monocytes did not express CCR2 in both the MDR-TB and DS-TB groups.

Together, these data indicate that CCR4 expression in peripheral blood monocytes is a distinctive immunological readout of MDR-TB. Although this receptor is mainly implicated in memory T cell traffic, our results may suggest that monocytes may draw on alternative chemotactic axes to infiltrate the sites of infection during MDR-TB. In contrast, depletion of CCR2+ monocytes is a feature shared by DS-TB and MDR-TB. Thus, our data support the hypothesis that leukocyte recruitment dynamics could be different in MDR-TB and DS-TB.

### 3.4. Naïve and Memory T Cell Subpopulations Are Differentially Regulated during MDR-TB

T cells play a pivotal role in establishing protective immune responses against Mtb. This function is mediated by different T cell subpopulations exerting activities like cytokine/chemokine production (helper CD4+ T cells) and lysis of infected cells (cytotoxic CD8+ T cells). The initiation and maintenance of protective adaptive responses depend on antigen presentation and naïve T cells' priming by phagocytes. This crucial process allows the expansion of several clones of antigen-specific lymphocytes and the development of memory T cell subpopulations that sustain long-lasting immunity against TB. Most of what is currently known about the kinetics of anti-Mtb T cell-mediated immunity comes from DS-TB studies. However, to what extend protective mechanisms against Mtb are dysregulated during MDR-TB is not well understood so far.

**Figure 4:**
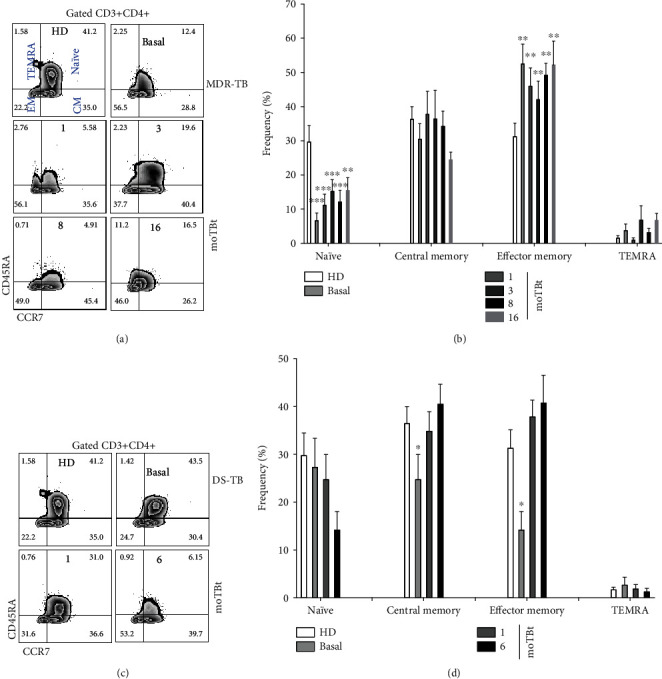
Kinetics of CD4+ T cell subpopulations in MDR-TB patients. Representative zebra plots showing the strategy of analysis used to enumerate naïve (CD45RA+CCR7+), central memory (CM, CD45RA-CCR7+), effector memory (EM, CD45RA-CCR7-), and TEMRA (CD45RA+CCR7-) CD3+CD4+ T cells in peripheral blood mononuclear cells (PBMCs) from multidrug-resistant (MDR-TB) and drug-sensitive (DS-TB) patients (a and c, respectively). (b) Frequency of CD3+CD4+ T cell subpopulations in MDR-TB patients (HD: *n* = 6, MDR-TB: basal, 1, and 3 moTBt *n* = 10, 8 moTBt *n* = 8, and 16 moTBt *n* = 7). (d) Frequency of CD3+CD4+ T cell subpopulations in DS-TB patients (HD *n* = 6, DS-TB: basal and 1 moTBt *n* = 10, and 6 moTBt n = 8). Bar graphs depict means ± standard error of the mean (SEM). Differences between patient groups and HD at each time point were analyzed using multiple Student's *t*-test corrected for multiple comparisons using the Sidak–Bonferroni method. ^∗^*p* < 0.05, ^∗∗^*p* < 0.01, ^∗∗∗^*p* < 0.001, and ^∗∗∗∗^*p* < 0.0001. moTBt: months of antituberculosis treatment.

**Figure 5:**
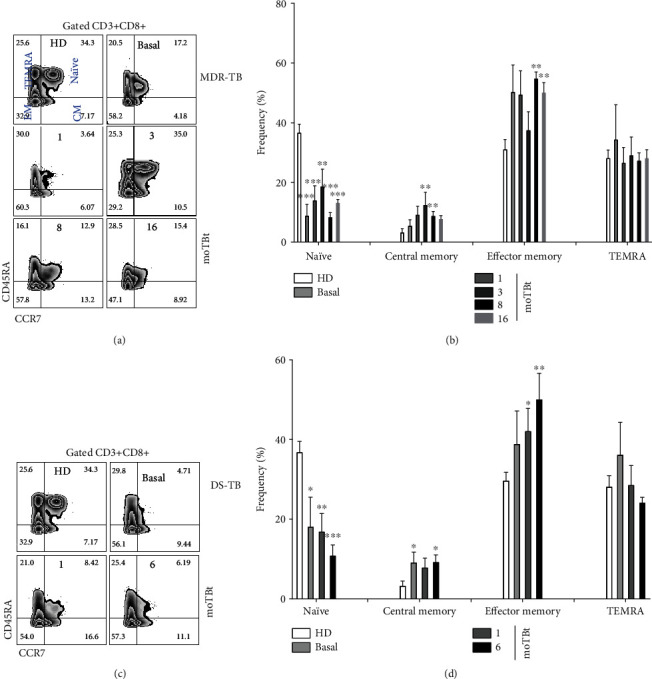
Kinetics of CD8+ T cell subpopulations in MDR-TB patients. Representative zebra plots showing the strategy of analysis used to enumerate naïve (CD45RA+CCR7+), central memory (CM, CD45RA-CCR7+), effector memory (EM, CD45RA-CCR7-), and TEMRA (CD45RA+CCR7-) CD3+CD8+ T cells in peripheral blood mononuclear cells (PBMCs) from multidrug-resistant (MDR-TB) and drug-sensitive (DS-TB) patients (a and c, respectively). (b) Frequency of CD3+CD8+ T cell subpopulations in MDR-TB patients (HD: *n* = 6, MDR-TB: basal, 1, and 3 moTBt *n* = 10, 8 moTBt *n* = 8, and 16 moTBt *n* = 7). (d) Frequency of CD3+CD8+ T cell subpopulations in DS-TB patients (HD *n* = 6, DS-TB: basal and 1 moTBt *n* = 10, and 6 moTBt *n* = 8). Bar graphs depict means ± standard error of the mean (SEM). Differences between patient groups and HD at each time point were analyzed using multiple Student's *t*-test corrected for multiple comparisons using the Sidak–Bonferroni method. ^∗^*p* < 0.05, ^∗∗^*p* < 0.01, ^∗∗∗^*p* < 0.001, and ^∗∗∗∗^*p* < 0.0001. moTBt: months of antituberculosis treatment.

Hence, we evaluated the phenotype and dynamics of T cell subpopulations in patients with DS-TB and MDR-TB. Notably, we did not find changes in total T cell frequencies (CD4+/CD8+ ratio) between groups (Supplementary Figure S3). We next focused on enumerating different CD4+ and CD8+ T cell subsets identified by a variable expression of the markers CD45RA and C-C chemokine receptor 7 (CCR7) [30, 31]. As such, we identified naïve (CD45RA+CCR7+), central memory (CM, CD45RA-CCR7+), effector memory (EM, CD45RA-CCR7-), and terminal effector CD45RA+ (TEMRA, CD45RA+CCR7-) helper and cytotoxic T cells (Figures 4 and 5, respectively).

Our analysis showed that MDR-TB patients have a decreased frequency of naïve CD4+ T cells, but high numbers of EM CD4+ T cells compared to HD. Interestingly, this phenotype was not modified by anti-TB treatment at 16 moTBt (Figure 4(b)). Conversely, DS-TB was characterized by decreased CM and EM CD4+ T cells at baseline, which recovered at 1 moTBt. These DS-TB changes were accompanied by a progressive decrease in the frequency of naïve CD4+ T cells, which was not statistically significant than HD (Figure 4(d)).

Regarding CD8+ T cells, both MDR-TB and DS-TB showed a depletion of naïve cells compared to HD. Also, both groups were characterized by a rise in CM CD8+ T cells that normalized only in MDR-TB patients at 16 moTBt. Finally, both MDR-TB and DS-TB have an increased frequency of the EM CD8+ T cells that was not modified by treatment, but this change occurred earlier in DS-TB at 1moTBt than in MDR-TB at 8 moTBt (Figures 5(b) and 5(d)). TEMRA CD4+ and CD8+ T cells showed no distinctive profiles in both patient groups (Figures 4(b), 3(d), 5(b), and 5(d)). Collectively, our results demonstrate that EM CD4+ T cells and CM/EM CD8+ T cells dominate the immune profile of MDR-TB. These findings may indicate a continuous stimulation of adaptive responses during MDR-TB that is not appeased by the anti-TB therapy, perhaps due to remaining subclinical Mtb activity in the lungs.

### 3.5. CXCR1+CXCR3+ T Cells Are Increased during MDR-TB and DS-TB

**Figure 6:**
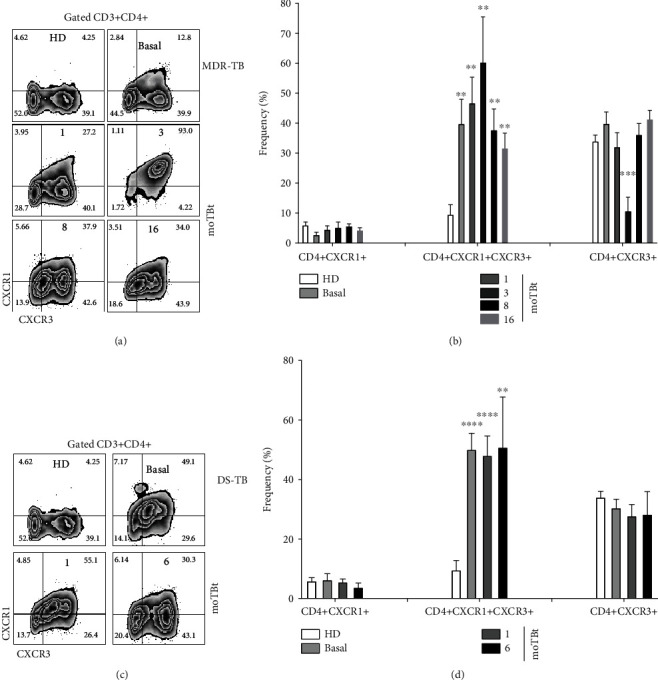
Expression of CXCR1 and CXCR3 in CD4+T cells from MDR-TB and DS-TB patients. Representative zebra plots showing the strategy used to determine the expression of CXCR1 and CXCR3 CD3+CD4+ T cells. Peripheral blood mononuclear cells (PBMCs) from multidrug-resistant (MDR-TB) or drug-sensitive (DS-TB) patients were obtained at different times before and after anti-TB treatment and used for phenotypical characterization by flow cytometry (a and c, respectively). (b) Frequency of CD4+CXCR1+, CD4+CXCR1+CXCR3+, and CD4+CXCR3+ T cell subpopulations in MDR-TB patients (HD: *n* = 6, MDR-TB: basal, 1, and 3 moTBt *n* = 10, 8 moTBt *n* = 8, and 16 moTBt *n* = 7). (d) Frequency CD4+CXCR1+, CD4+CXCR1+CXCR3+, and CD4+CXCR3+ T cell subpopulations in DS-TB patients (HD: *n* = 6, DS-TB: basal and 1 moTBt *n* = 10, and 6 moTBt *n* = 8). Bar graphs show means ± standard error of the mean (SEM). Differences between patient groups and HD at each time point were analyzed using multiple Student's *t*-test corrected for multiple comparisons using the Sidak–Bonferroni method. ^∗^*p* < 0.05, ^∗∗^*p* < 0.01, ^∗∗∗^*p* < 0.001, and ^∗∗∗∗^*p* < 0.0001. moTBt: months of antituberculosis treatment.

**Figure 7:**
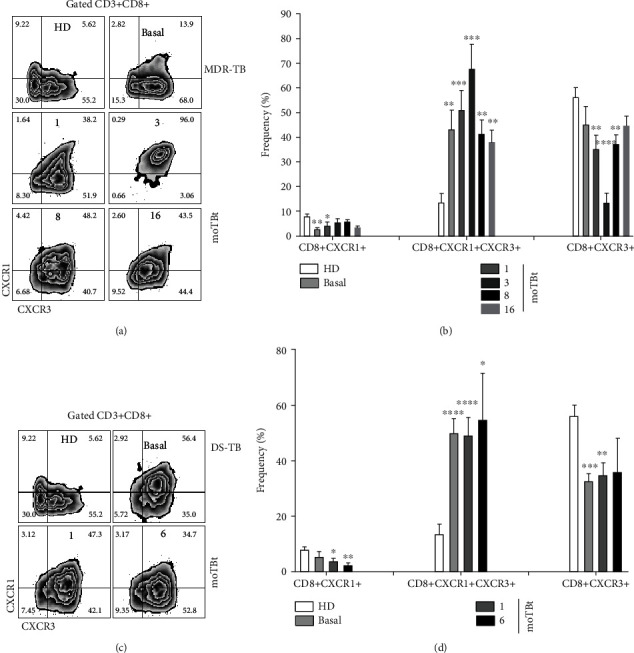
Expression of CXCR1 and CXCR3 in CD8+T cells from MDR-TB and DS-TB patients. Representative zebra plots showing the strategy used to determine the expression of CXCR1 and CXCR3 CD3+CD8+ T cells. Peripheral blood mononuclear cells (PBMCs) from multidrug-resistant (MDR-TB) or drug-sensitive (DS-TB) patients were obtained at different times before and after anti-TB treatment and used for phenotypical characterization by flow cytometry (a and c, respectively). (b) Frequency of CD8+CXCR1+, CD8+CXCR1+CXCR3+, and CD8+CXCR3+ T cell subpopulations in MDR-TB patients (HD: *n* = 6, MDR-TB: basal, 1, and 3 moTBt *n* = 10, 8 moTBt *n* = 8, and 16 moTBt *n* = 7). (d) Frequency CD8+CXCR1+, CD8+CXCR1+CXCR3+, and CD8+CXCR3+ T cell subpopulations in DS-TB patients (HD: *n* = 6, DS-TB: basal and 1 moTBt *n* = 10, and 6 moTBt *n* = 8). Bar graphs show means ± standard error of the mean (SEM). Differences between patient groups and HD at each time point were analyzed using multiple Student's *t*-test corrected for multiple comparisons using the Sidak–Bonferroni method. ^∗^*p* < 0.05, ^∗∗^*p* < 0.01, ^∗∗∗^*p* < 0.001, and ^∗∗∗∗^*p* < 0.0001. moTBt: months of antituberculosis treatment.

The receptors CXCR1 and CXCR3 play essential functions during the immune response against TB [30]. Although these receptors are preferentially expressed in neutrophils from TB patients, some reports showed that CXCR1 and CXCR2 could also be expressed on the cell surface of effector CD4+ and CD8+ T cells [31, 32]. Here, we addressed possible differences in the expression of CXCR1 and CXCR3 in CD4+ and CD8+ T cells from DS-TB and MDR-TB patients (Figures 6 and 7, respectively).

We observed that, compared with HD, MDR-TB and DS-TB patients have an increased frequency of double-positive CXCR1+CXCR3+ CD4+ T cells at baseline. Interestingly, the frequency of this subpopulation did not return to normal levels by the end of the anti-TB treatment. Frequencies of single-positive CXCR1+ and CXC3+ CD4+ T cells in MDR-TB and DS-TB patients were similar to HD (Figures 6(b) and 6(d)). The double-positive CXCR1+CXCR3+ CD8+ T cell subpopulation was also found elevated at all follow-up time points in DS-TB and MDR-TB with respect to HD (Figures 7(b) and 7(d)). Meanwhile, CXCR1+CD8+ T cells showed a decrease at baseline but an early recovery at 3 moTBt in MDR-TB. In contrast, DS-TB patients exhibited a progressive decline in CXCR1+CD8+ T cells that did not normalize after anti-TB treatment (Figures 7(b) and 7(d)). CXCR3+CD8+ T cells were depleted from the circulation in MDR-TB patients at 3 and 8 moTBt. Similarly, in DS-TB, this subpopulation was decreased at baseline and slightly recovered progressively by the end of anti-TB therapy (Figures 7(b) and 7(d)).

Based on our results, we propose that efficient recruitment of CD4+ and CD8+ T cells during DS-TB and MDR-TB requires the concomitant expression of CXCR1 and CXCR3. As such, double-positive CXCR1+CXCR3+ may improve their capacity to respond to a broader range of chemotactic signals than single-positive cells.

### 3.6. Migration Capacity of Leukocytes from MDR-TB and DS-TB Patients

**Figure 8:**
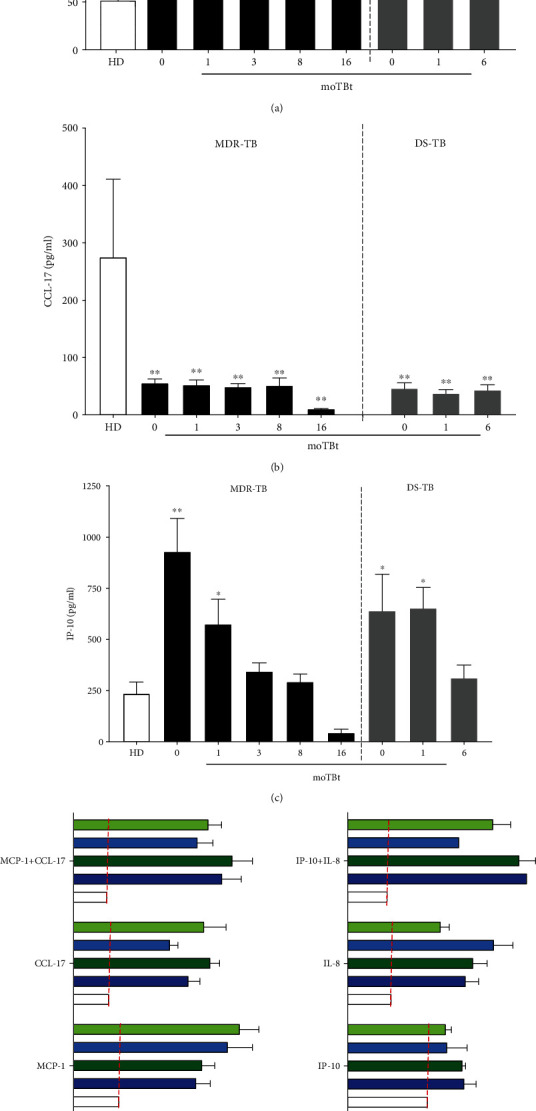
Plasma chemokine levels and migration capacity of leukocytes during MDR-TB and DS-TB. Plasma levels of (a) MCP-1, (b) CCL-17, and (c) IP-10 were determined by ELISA in multidrug-resistant (MDR-TB) and drug-sensitive (DS-TB) patients. (HD: *n* = 6, MDR-TB: basal, 1, and 3 moTBt *n* = 14, 8 moTBt *n* = 11, and 16 moTBt *n* = 10. DS-TB: basal and 1 moTBt and 6 moTBt *n* = 11). Peripheral blood mononuclear cells (PBMCs) from DS-TB and MDR-TB patients isolated before and after anti-TB treatment, evaluated for their ability to migrate in response to (d) MCP-1 and CCL17 or (e) IP-10 and IL-8, using a fluorometric assay (HD: *n* = 3, MDR-TB: *n* = 4 (before treatment) and *n* = 3 (after treatment), DS-TB: *n* = 3). The relative fluorescence unit (RFU) is indicative of the magnitude of cellular migration. Bar graphs show means ± standard error of the mean (SEM). Differences between patient groups and HD at each time point or experimental condition were analyzed using multiple Student's *t*-test corrected for multiple comparisons using the Sidak–Bonferroni method. ^∗^*p* < 0.05, ^∗∗^*p* < 0.01, ^∗∗∗^*p* < 0.001, and ^∗∗∗∗^*p* < 0.0001. moTBt: months of antituberculosis treatment.

As aforementioned, the migration capacity of immune cells depends on their chemokine receptor expression profile and the magnitude and dynamics of chemokine production. Our previous results showed important changes in the phenotype of chemokine receptor expression in monocytes and T cells during MDR-TB. To further explore the regulation of leukocyte traffic during this disease, we measured plasma levels of MCP-1, CCL-17, IL-8, and IP-10, ligands of CCR2, CCR4, CXCR1, and CXCR3 receptors, respectively, in patients with MDR-TB and DS-TB (Figures 8(a)–8(c)).

Our data showed that MCP-1 is highly released to the plasma of MDR-TB and DS-TB patients compared to HD. Interestingly, plasma MCP-1 levels continued to elevate during both diseases, even at the end of chemotherapy (Figure 8(a)). Meanwhile, plasma CCL-17 levels were decreased during MDR-TB and DS-TB and did not recover after the end of treatment (Figure 8(b)). Strikingly, MDR-TB and DS-TB exhibited high levels of IP-10 at baseline and 1 moTBt. However, IP-10 progressively decreased from the third moTBt to the end of treatment in MDR-TB patients and at the end of therapy in DS-TB individuals (Figure 8(c)). Plasma IL-8 levels were under the limit of detection in all participant groups (data not shown). These findings indicate that possible differences in leukocyte migration during MDR-TB and DS-TB are conditioned by chemokine receptors' expression but not plasma levels of soluble chemotactic factors. As such, we observed changes in the expression of CCR2 and CCR4 in monocytes from MDR-TB and DS-TB patients, but their chemokine ligands' plasma levels were similar between groups.

Next, we addressed whether MDR-TB induced different immune cell recruitment patterns than DS-TB. For this purpose, we conducted *in vitro* migration assays using PBMCs exposed to MCP-1 (100 ng/ml), CCL-17 (20 ng/ml), MCP-1+CCL-17, IP-10 (50 ng/ml), IL-8 (50 ng/ml), and IP-10+IL-8 (Figures 8(d) and 8(e)). Overall, cells from TB patients have a higher ability to migrate than HD cells. Indeed, PBMCs from MDR-TB and DS-TB patients obtained before and after anti-TB treatment showed a 2-fold increase in MCP-1-induced migration. Similar increases in cellular migration in response to CCL-17 were observed in both participant groups, although MDR-TB patients showed a slight reduction in migration at the end of treatment compared to DS-TB individuals (Figure 8(d)). PBMCs from MDR-TB and DS-TB patients highly responded to IP-10 as well, showing a 50% increased migration ability compared to HD. This feature was discretely reduced by anti-TB treatment. Robust responses to IL-8 were also identified, although DS-TB, but not MDR-TB patients, showed a 50% reduction in migration after treatment (Figure 8(e)).

Remarkably, CD11b+CD3+ myeloid cells from DS-TB, but not MDR-TB patients, highly responded to MCP-1 and CCL-17 (Supplementary Figure S4). This was surprising because CD11b+CD3+ cells poorly expressed CCR2 contrary to classic monocytes (CD14+CD3-CD16-). Hence, CD11b+CD3+ cells may respond to MCP-1 via CCR2-independent mechanisms. Interestingly, CCR2 was also expressed at high levels in the second proinflammatory monocyte subpopulation (CD14+CD3-CD16+) of DS-TB patients. This finding suggests that specific monocyte subpopulations possess different migration profiles (Supplementary Figure S4). Finally, IP-10 induced strong migration responses only in CD8+ T cells from DS-TB (Supplementary Figure S4).

## 4. Discussion

This study identified changes in the distribution of several immune cell subpopulations, expression of chemokine receptors, chemokine plasma levels, and migration capacity in MDR-TB and DS-TB patients. Our results evidence a specific immune profile in MDR-TB patients that is not regulated by anti-TB treatment and is different from DS-TB, even when both groups had similar demographic data, including diabetes mellitus as the most prevalent comorbidity. Moreover, both TB groups revealed similarities in lung parenchymal damage, although fibrotic lesions predominated in MDR-TB patients due to the disease's chronicity (Supplementary Figure S5 and Table S2). Together, these results allow to suggest that the differences observed here may contribute to a delayed control of MDR-TB.

Monocytes are the primary precursor of macrophages in tissues. The expression of CD16 characterizes a proinflammatory monocyte subset that secretes a high amount of proinflammatory cytokines. However, it was recently reported that CD3+ macrophages could also deliver proinflammatory cytokines [12, 33]. Interestingly, we found that both MDR-TB and DS-TB patients have an increased frequency of nonclassical monocytes, such as CD14+CD3-CD16+ and CD14+CD3+CD16-. Nonetheless, only CD14+CD3-CD16+ monocytes in DS-TB exhibit a high expression of CCR2, a proinflammatory marker. In consonance, recently, we reported that MDR-TB patients exhibit a proinflammatory cytokine profile for a longer time than DS-TB, and this status could be related to an increased frequency of HLA-II+CD16+ monocytes [34]. These together suggest that MDR-TB patients have increased nonclassical monocytes' frequency, which could be responsible for favoring a systemic inflammatory status.

Our study does not clarify whether the decrease in the frequency of blood proinflammatory monocytes observed in response to anti-TB treatment is due to a switch from the proinflammatory to immune regulatory response or results from the migration of immune cells to local infection sites. Previous reports demonstrated that classical monocytes confer protection against Mtb and enhance their migration capacity in response to mycobacterial soluble components, suggesting that these monocytes can mediate an intense pulmonary infiltration, decreasing the frequency of this subpopulation in blood [6, 35].

Other nonclassic monocytes have also been subdivided by CD16 expression, and the authors suggested that CD14+CD16+ monocytes have a low expression of CCR2 in the early stages of Mtb infection. The expression of other molecules, such as sphingolipid GM1, correlates with functional differences in endocytic activity and susceptibility criteria to mycobacterial infection and response to lipopolysaccharides [36, 37]. Our data is in agreement with the low CCR2 expression on the cell surface of proinflammatory monocytes. The accumulation of this monocyte subtype in circulating blood could be due to this low CCR2 expression.

Recently, we demonstrated that CD3+ myeloid cells accumulated in the liver of mice with systemic infection of BCG. These cells can secrete proinflammatory cytokines and can also respond to them. The maintenance of this leukocyte subpopulation requires the expression of TNF receptor 1 (TNFR1) [12, 13]. As expected, the frequency of circulating CD3+ monocytes was increased in both MDR-TB and DS-TB patients compared with HD. Surprisingly, CD3+ monocytes did not express CD16, indicating that they could constitute an independent proinflammatory population. The frequency of CD3+ monocytes decreases earlier in DS-TB than in MDR-TB during treatment.

Interestingly, even if CD3+ monocytes in DS-TB do not strongly express CCR2, CD3+ myeloid cells continue exhibiting a high ability to migrate *in vitro*. This could explain the decrease in the frequency of circulating CD3+ monocytes as they are recruited to the infection site by a CCR2-independent pathway to aid in the formation of granuloma and infection control. Our study opens new questions regarding CD3+ monocytes in the context of Mtb infection, including identifying the origin of the CD3+ macrophages. Considering the description in this study and those reported in the granuloma of TB patients, both could be differentiated directly from circulating CD3+ monocytes, but this has not been proven to date. Previously, it was demonstrated that recruited circulating monocytes are well-poised to influence multiple aspects of host immunity against Mtb infections in the lung. They can be differentiated into both macrophage dendritic cell lineages following mycobacterial infection [38].

Monocyte recruitment at the infection site has been described as a fundamental step to activate an efficient immune response in different contexts, including Mtb infection [39]. CCR2 expression occurred mainly in the classic monocyte subtype; however, nonclassic monocyte subsets demonstrated the ability to migrate, possibly using a CCR4-dependent pathway. The increased expression of this receptor observed here supports this idea. The role of CCR4 in inducing migration has been shown utilizing different disease models; for example, in prostate cancer, tumor-associated macrophages promote metastasis via the activation of the CCL2-CCR2 or the CCR4 axis, whereas circulating monocytes from patients with rheumatoid arthritis (RA) showed a high expression of CCR4, suggesting that CCR4-dependent migration is required for monocyte recruitment to the joint [40, 41].

CD4 : CD8 T cell ratios provide a simple parameter to indicate the balance between the two leading T cell subpopulations. These ratios are used frequently to monitor the progress of the infection in HIV+ patients. CD4 : CD8 ratios provide information on pathophysiological features and clinical implications [42, 43]. The CD4 : CD8 ratio was not modified in our cohort of TB patients, but when we evaluated the distribution of naïve, CM, EM, and TEMRA T cells, our data revealed that CD4+ T cell profiles are strongly modified in MDR-TB but not in DS-TB. The naïve subpopulation decreased by 50%, and it did not recover, even by the end of therapy, while the EM population increased by nearly 40%. The high prevalence of memory cells is a relevant difference between MDR-TB and DS-TB patients, due to that memory cells can produce high amounts of cytokines and proinflammatory chemokines, which is an undesirable adverse effect that may favor long-term immune activation [44]. At present, the effects of long-term activation and chronic inflammation are better understood in the context of chronic diseases such as AIDS, TB, and cancer. In these diseases, T cells have shown defects in cytokine production and cytotoxicity, and they are not able to replicate in response to antigenic stimuli [45–48]. A hallmark of induced long-term exhaustion and senescence comprises a higher expression of exhaustion-associated receptors, such as TIM-3, PD-1, KLRG1, and CTLA-4 [45, 49–51]. It has been reported that HIV+ patients who develop tuberculosis-associated immune reconstitution inflammatory syndrome (TB-IRIS) as a result of long-term activation present a higher number of exhausted cells (PD-1+, KLRG-1+) that are not effective in combating the TB infection [46]. The exhausted cells are generated by a continuous antigenic-activation process associated with a long-term proinflammatory process in HIV-TB coinfection. Thus, CD4+ T cell subpopulations could also be regulated differently in MDR-TB vs. DS-TB. The immune response led by CD4+ lymphocytes may be disturbed in the presence of MDR-TB strains; consequently, the recovery time of MDR-TB and DS-TB exhausted cells could be different.

Interestingly, although we found that the frequency of CD8 T cell distribution is similar in both the MDR-TB and the DS-TB groups, we observed a decrease in the levels of the naïve subpopulation and an increase in the EM subpopulation in both TB groups, suggesting that drug-resistant status does not affect the distribution of the CD8+ T cells. Recently, it was reported that CD8+ T cells in DS-TB exhibit higher cytotoxicity, which is mediated by the HLA-I classical pathway [52]. The latter effect could be partially explained by the increase of memory cells that we observed. This is despite that our data showed that CD8+ T cell subpopulations have similar distributions in both MDR-TB and DS-TB, suggesting that it is possible that high cytotoxicity capacity is typical in TB and is independent of the antibiotic resistance.

Our data suggest that TB patients have an accumulation of CD4+ and CD8+ cells that coexpress CXCR1 and CXCR3 and increased serum levels of IL-8 and IP-10. Recently, using competitive adoptive transfer migration assays and mathematical modeling, it was reported that Mtb-specific CD4 T cells migrate from the blood to the lung parenchyma during 8.6 h after adoptive transference, whereas the CXCR3 and CX3CR1 deficiency decreases the average rate of Th1 cell entry into the lung parenchyma. Additionally, CCR2 and CXCR5 exhibited a lesser degree of influence. This report suggested that T cells' recruitment to the lungs in Mtb infections is regulated by the cumulative effects of multiple chemokine receptors that promote or inhibit entry into the lung parenchyma [53]. Our study provides data to support the hypothesis that cellular recruitment is a mechanism that is orchestrated by a balance between chemokine receptors and their microenvironment. Depending on this balance, the recruitment of specific cell subpopulations could be promoted or inhibited.

In summary, we demonstrated, to our knowledge for the first time, that the accumulation of circulating CD3+ monocytes is downregulated in blood from DS-TB patients by the end of anti-TB therapy, while MDR-TB patients only show a partial decrease at 16 moTBt. Interestingly, our data also demonstrated that CD11b+CD3+ entertains a high capacity for migration in DS-TB patients. Contrariwise, the frequency of CD11b+CD3+ that migrate in MDR-TB is lower. Regarding lymphocytes, CD4+ and CD8+ T cell migration only reveal slight changes in both MDR-TB and DS-TB. We suggest that regulation of the immune cellular subpopulation distribution is lower in MDR-TB than in DS-TB, at least in blood. This is probably helpful for better cellular accumulation at the infection site, as we previously reported in the murine model. Thus, our results not only describe a new idea, to our knowledge, of the pathogenesis of TB, mediated by the difference between drug-resistant Mtb strains, but also provide highlights in the search for novel therapeutic targets or markers for conducting follow-up in patients with TB.

## Figures and Tables

**Table 1:** Baseline characteristics of the study population.

Characteristic	HD (*n* = 6)	MDR-TB (*n* = 14)	DS-TB (*n* = 11)	*p* value (MDR vs. DS)
Age (years), median (IQR)	27 (19-40)	41 (36-47)	38 (27-52)	0.717
Male gender, *n* (%)	3 (50%)	12 (86%)	9 (81%)	0.604
DM2, *n* (%)	0 (0%)	10 (71%)	5 (45%)	0.183
Alcoholism, *n* (%)	0 (0%)	6 (43%)	2 (18%)	0.190
Smoking, *n* (%)	0 (0%)	3 (21%)	3 (27%)	0.548
BMI (kg/m^2^), median (IQR)	23 (21-26)	24 (18-25)	23 (20-25)	0.837
Previous TB treatment, *n* (%)	—	14 (100%)	1 (9%)	<0.005
Time of first TB diagnosis (years), median (IQR)	—	6 (2-8)	2∗	<0.005

Differences in categorical and continuous variables were analyzed with the Fisher exact test and Mann–Whitney *U* test, respectively. Values of *p* < 0.05 were considered significant. BMI: body mass index; DM2: type 2 diabetes mellitus; DS-TB: drug-susceptible tuberculosis; IQR: interquartile range (IQR); MDR-TB: multidrug-resistant tuberculosis. ^∗^Data from one patient.

**Table 2:** Laboratory characteristics of the study population.

Characteristic	Total (*n* = 25)	MDR-TB (*n* = 14)	DS-TB (*n* = 11)	*p* value (MDR vs. DS)
Leukocytes, median (IQR), (RV 4 − 10 × 10^3^ cells/mm^3^)	10 (9-11)	10 (8-11)	10 (9-11)	0.970
Neutrophils, median (IQR), (RV 2 − 7.5 × 10^3^ cells/mm^3^)	6.9 (6-8)	7 (5-7)	6.9 (6-8)	0.851
Lymphocytes, median (IQR), (RV 1-4 x 10^3^ cells/mm^3^)	1.5 (1.3-2.1)	1.5 (1.3-2.1)	1.5 (0.9-2)	0.548
Monocytes, median (IQR), (RV 0.2 − 1 × 10^3^ cells/mm^3^)	0.6 (0.4-0.9)	0.6 (0.5-0.8)	0.9 (0.3-0.9)	0.852
Platelets, median (IQR), (RV 150 − 500 × 10^3^ cells/mm^3^)	325 (276-362)	333 (383-427)	299 (270-325)	0.204
Hemoglobin, median (IQR), (RV 11.5-17 mg/dl)	13 (12-14)	14 (12-16)	12.6 (12-14)	0.455
Glucose, median (IQR), (RV 74-106 mg/dl)	134 (96-150)	117 (87-150)	138 (107-229)	0.262
Albumin, median (IQR), (RV 3.5-4.8 mg/dl)	3.5 (2.8-3.8)	3.6 (3.1-3.8)	2.8 (2.6-3.5)	0.117
LDH, median (IQR), (RV 98-192 U/l)	158 (136-196)	155 (141-194)	183 (119-198)	0.868

Mann–Whitney *U* test *p* values < 0.05 were considered as significant. LDH: lactate dehydrogenase; DS-TB: drug-susceptible tuberculosis; IQR: interquartile range; MDR-TB: multidrug-resistant tuberculosis. RV: reference value (provided by the institutional Clinical Laboratory).

## Data Availability

The authors confirm that the raw data to support the conclusions of this study are included in the manuscript. The corresponding author will provide more information, upon reasonable request, to any qualified researcher.

## Abbreviations

AIDS: Acquired immune deficiency syndrome
BCG: *Mycobacterium bovis* bacillus Calmette-Guerin
CCL-17: C-C motif chemokine ligand 17
CCR2: Chemokine receptors C-C-motif chemokine receptor type 2
CCR4: Chemokine receptors C-C motif chemokine receptor type 4
CD: Cluster of differentiation
CM: Central memory
CTLA-4: Cytotoxic T-lymphocyte antigen 4
CXCR1: C-X-C motif chemokine receptor 1
CXCR3: C-X-C motif chemokine receptor 3
DM2: Type 2 diabetes mellitus
DS-TB: Drug-sensitive tuberculosis
EM: Effector memory
FMI: Fluorescence mean intensity
FMO: Fluorescence minus one
HD: Healthy donors
HIV: Human immunodeficiency virus
IFN: Interferon
IL: Interleukin
IP-10: Interferon *γ*-induced protein 10
IQR: Interquartile range
KLRG-1: Killer cell lectin-like receptor subfamily G member 1
LHCC: Local health care centers
MCP-1: Monocyte chemoattractant protein-1
MDR-TB: Multidrug-resistant tuberculosis
moTBt: Months after anti-TB treatment
PBMC: Peripheral blood mononuclear cells
PD-1: Programmed cell death 1
SEM: Standard error of the mean
TB: Tuberculosis
TEMRA: Terminal effector RA+
TIM-3: T cell immunoglobulin and mucin domain-3
TNF: Tumor necrosis factor
TNFR1: TNF receptor 1
WHO: World Health Organization.

## Supplementary Materials

Supplementary Materials We have attached a file with additional data sets such as graphs and tables to provide complementary information of interest.
