# Supplementary material for: Leukocytes from Patients with Drug-Sensitive and Multidrug-Resistant Tuberculosis Exhibit Distinctive Profiles of Chemokine Receptor Expression and Migration Capacity
Source: J Immunol Res. 2021 Apr 21;2021:6654220. doi: 10.1155/2021/6654220 (PMC8084684; doi:10.1155/2021/6654220)
Supplement: Supplementary Materials — We have attached a file with additional data sets such as graphs and tables to provide complementary information of interest. [file 6654220.f1.zip › MS Chemokines migration S4 .pptx]

## Slide 1
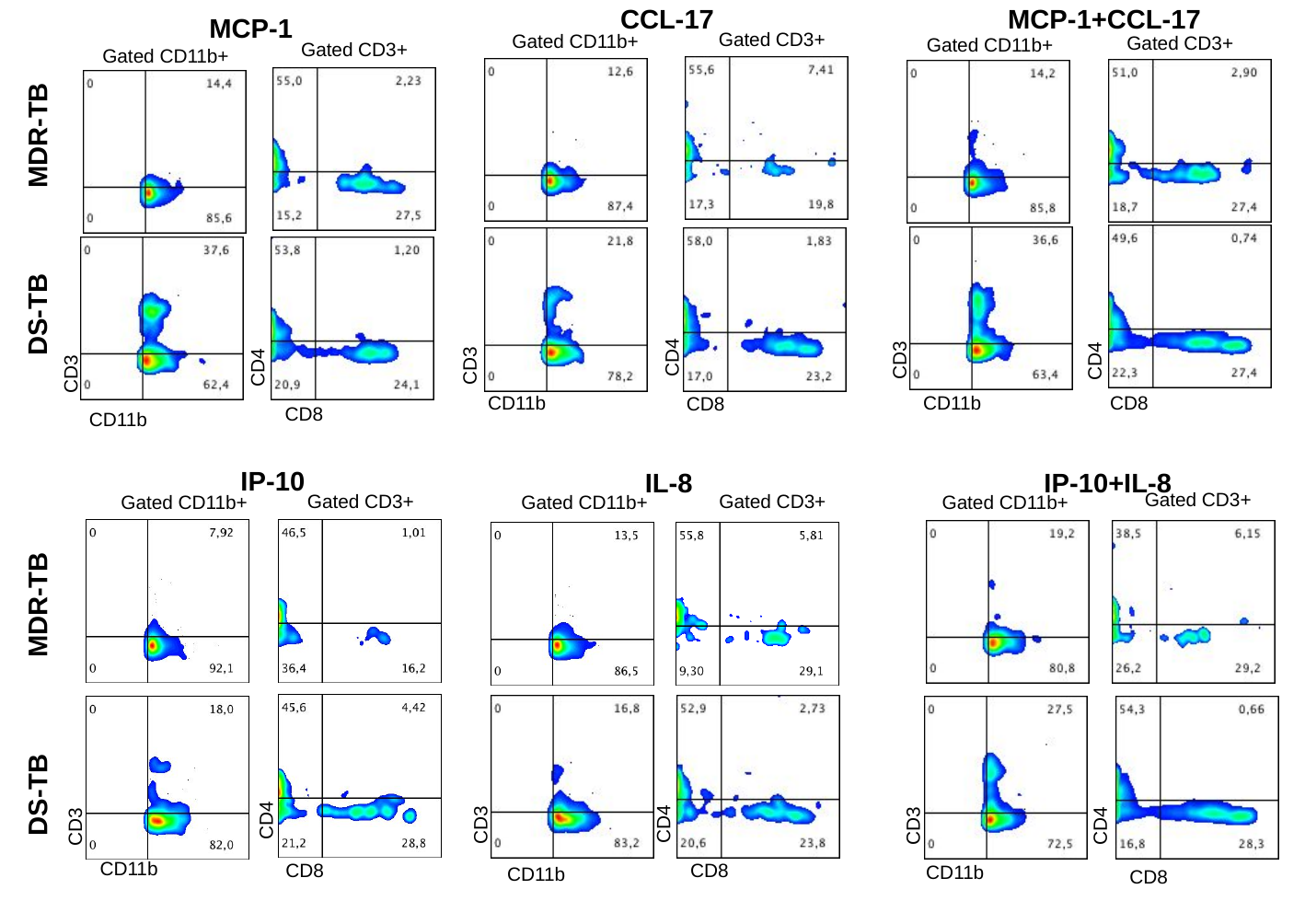

CCL-17
MCP-1+CCL-17
MCP-1
Gated CD3+
Gated CD11b+
Gated CD3+
Gated CD11b+
Gated CD3+
Gated CD11b+
MDR-TB
DS-TB
CD4
CD3
CD4
CD3
CD4
CD3
CD11b
CD8
CD11b
CD8
CD8
CD11b
IP-10
IP-10+IL-8
IL-8
Gated CD3+
Gated CD3+
Gated CD3+
Gated CD11b+
Gated CD11b+
Gated CD11b+
MDR-TB
DS-TB
CD4
CD4
CD3
CD3
CD4
CD3
CD11b
CD8
CD8
CD11b
CD11b
CD8
