# Supplementary figures and images for: Leukocytes from Patients with Drug-Sensitive and Multidrug-Resistant Tuberculosis Exhibit Distinctive Profiles of Chemokine Receptor Expression and Migration Capacity
Source: J Immunol Res. 2021 Apr 21;2021:6654220. doi: 10.1155/2021/6654220 (PMC8084684; doi:10.1155/2021/6654220)

## Slide 1
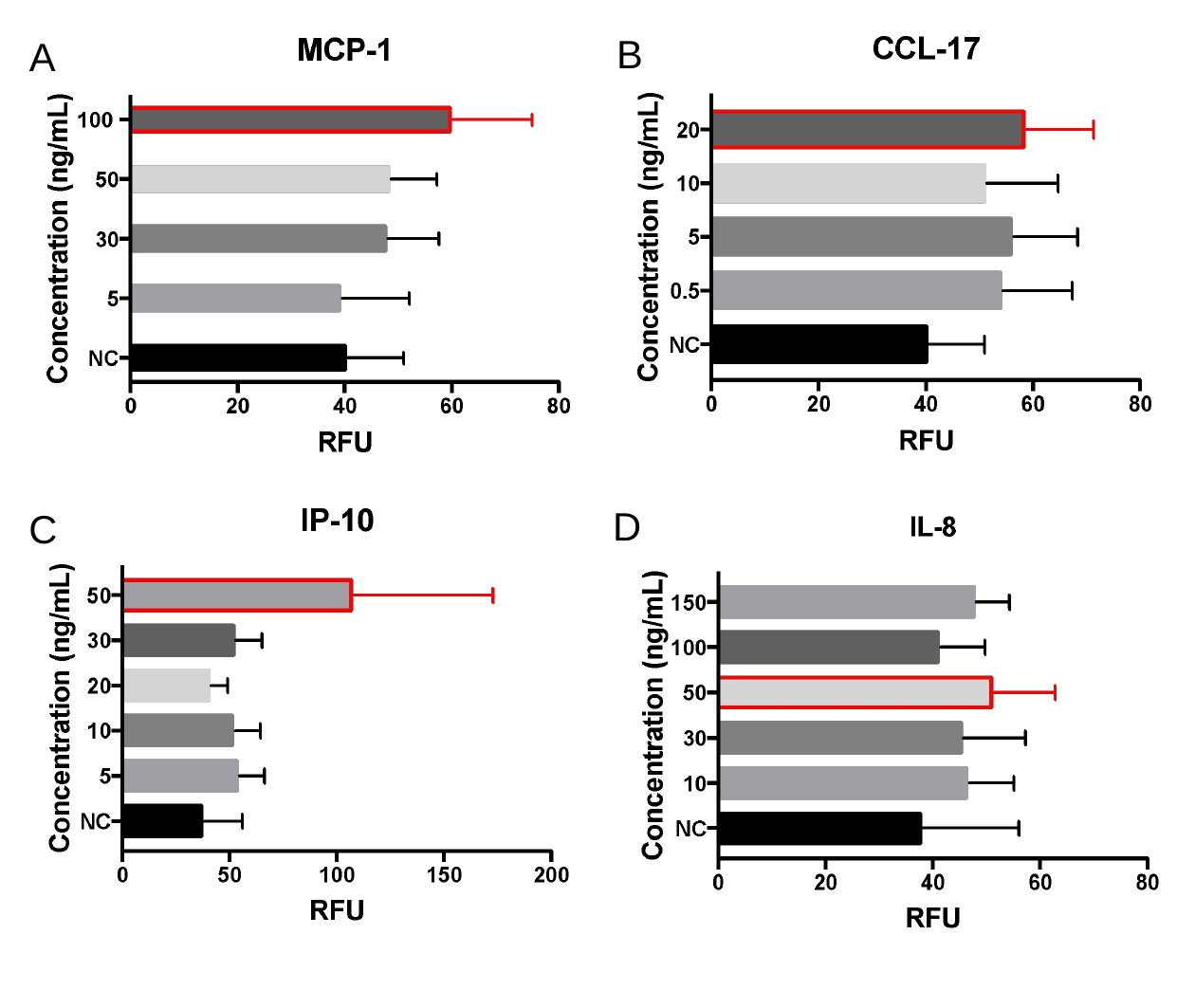

B
A
D
C

Supplement: Supplementary Materials — We have attached a file with additional data sets such as graphs and tables to provide complementary information of interest. [file 6654220.f1.zip › MS Chemokines migration S1 pptx.pptx]

## Slide 1
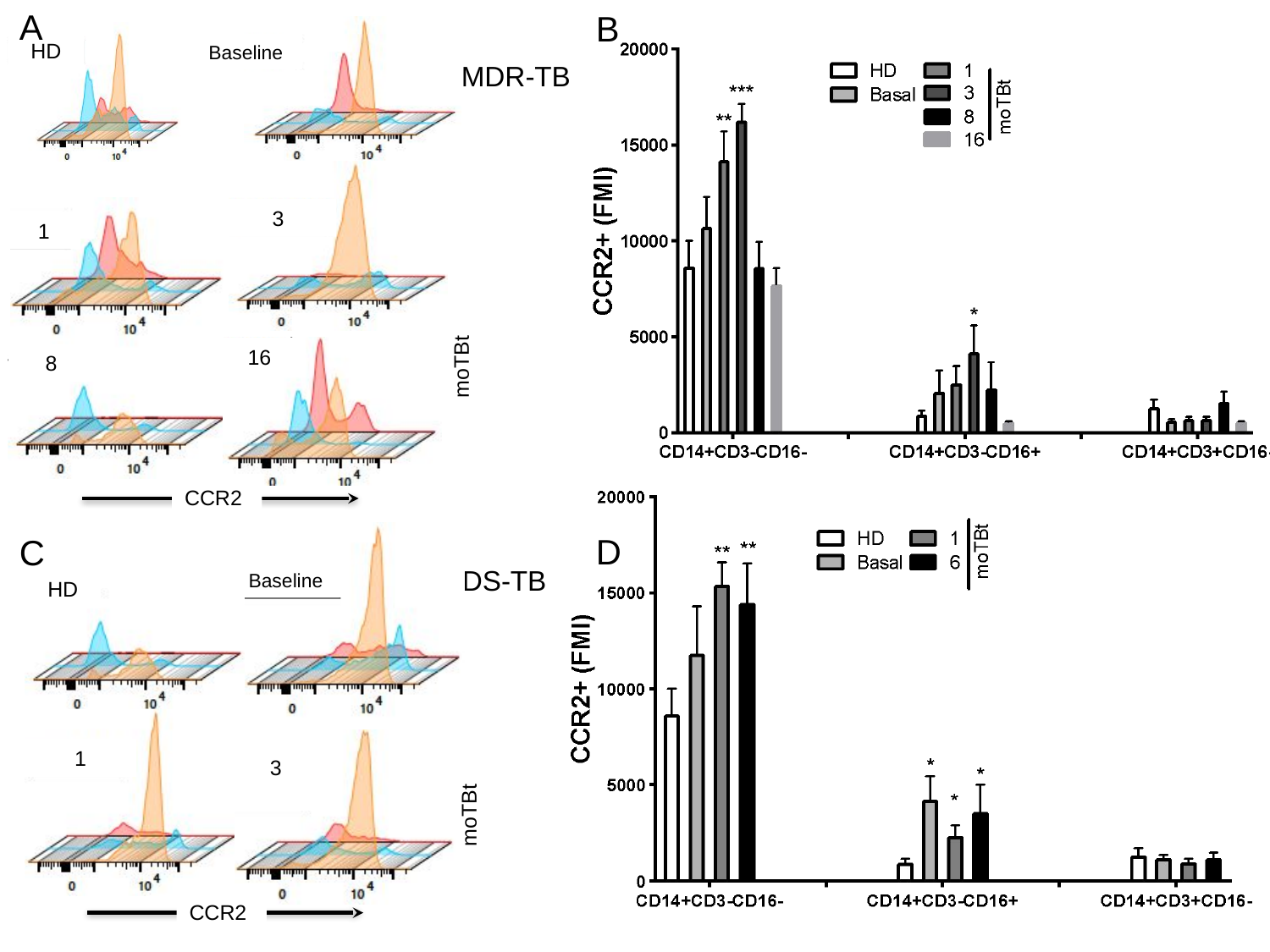

B
A
HD
Baseline
3
1
16
8
CCR2
MDR-TB
moTBt
Baseline
HD
1
3
moTBt
CCR2
D
C
DS-TB

Supplement: Supplementary Materials — We have attached a file with additional data sets such as graphs and tables to provide complementary information of interest. [file 6654220.f1.zip › MS Chemokines migration S2.pptx]

## Slide 1
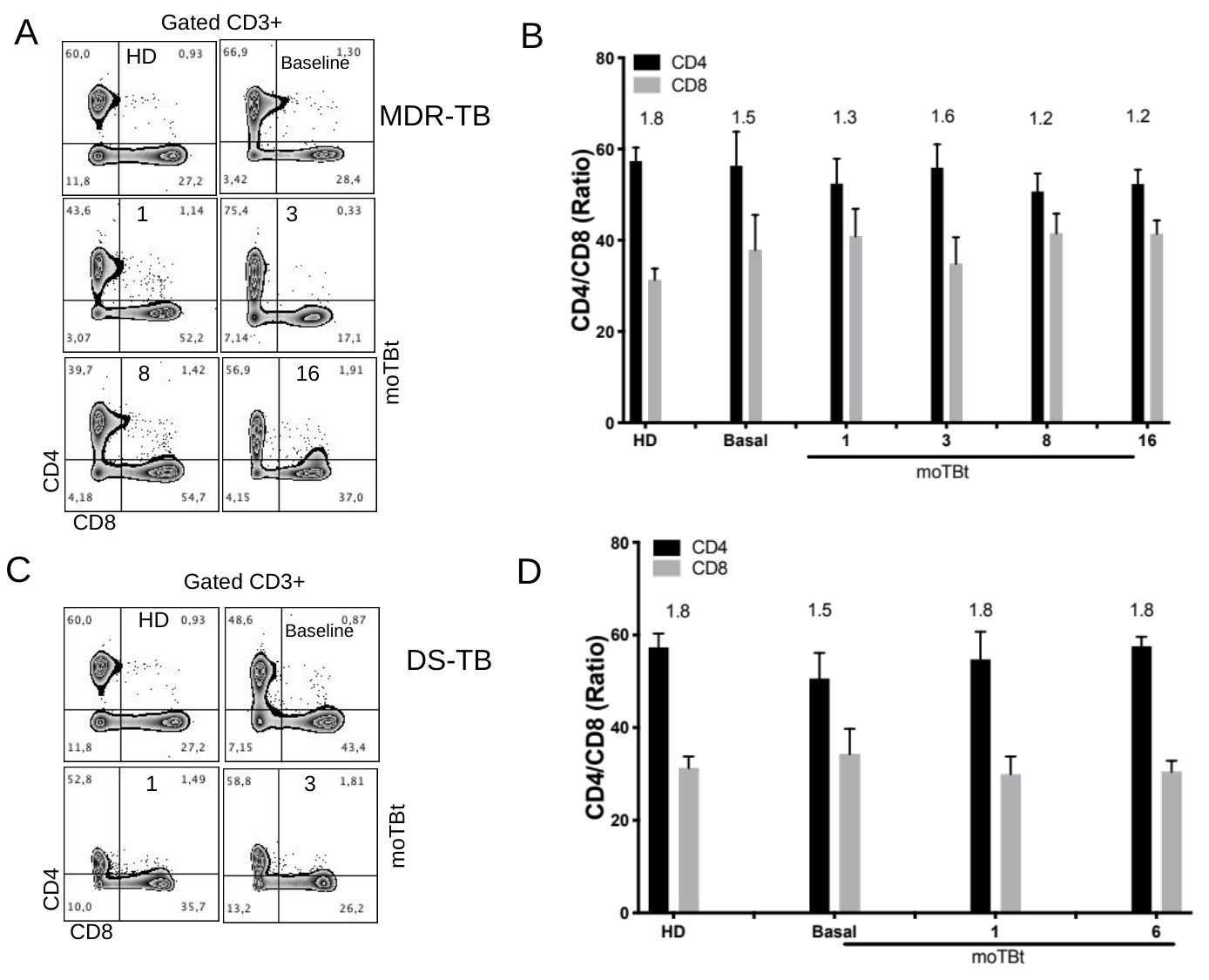

Gated CD3+
HD
Baseline
MDR-TB
1
3
8
moTBt
16
CD4
CD8
A
B
C
D
Gated CD3+
HD
Baseline
DS-TB
1
3
moTBt
CD4
CD8

Supplement: Supplementary Materials — We have attached a file with additional data sets such as graphs and tables to provide complementary information of interest. [file 6654220.f1.zip › MS Chemokines migration S3 .pptx]

## Slide 1
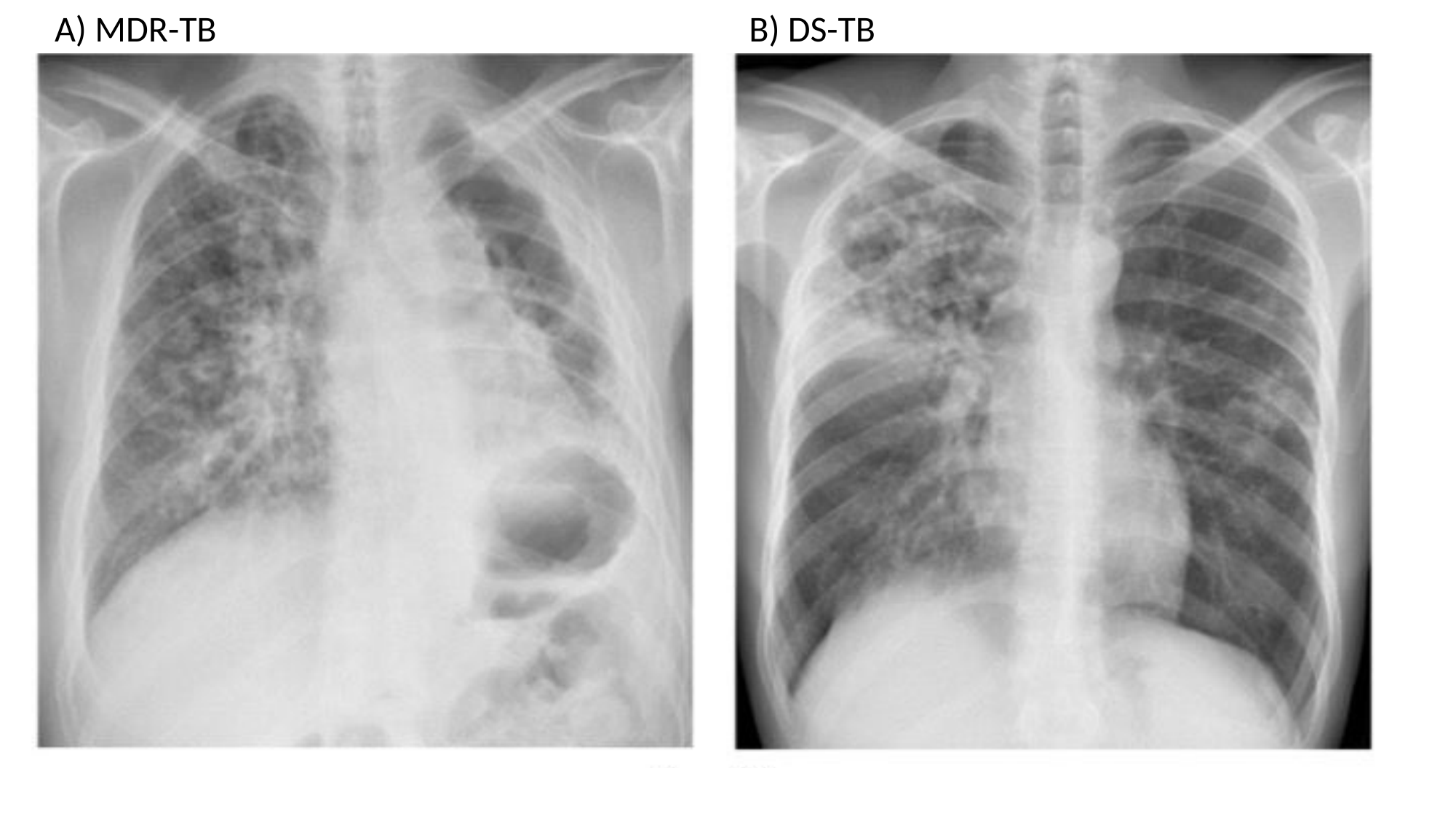

A) MDR-TB B) DS-TB

Supplement: Supplementary Materials — We have attached a file with additional data sets such as graphs and tables to provide complementary information of interest. [file 6654220.f1.zip › MS Chemokines migration S5 .pptx]
